# Increasing Accessibility to the Blind of Virtual Environments, Using a Virtual Mobility Aid Based On the "EyeCane": Feasibility Study

**DOI:** 10.1371/journal.pone.0072555

**Published:** 2013-08-19

**Authors:** Shachar Maidenbaum, Shelly Levy-Tzedek, Daniel-Robert Chebat, Amir Amedi

**Affiliations:** 1 Department of Medical Neurobiology, Institute for Medical Research Israel-Canada, Faculty of Medicine, Hebrew University of Jerusalem, Hadassah Ein-Kerem, Jerusalem, Israel; 2 The Edmond and Lily Safra Center for Brain Research, the Hebrew University of Jerusalem, Hadassah Ein-Kerem, Jerusalem, Israel; UC Davis School of Medicine, United States of America

## Abstract

Virtual worlds and environments are becoming an increasingly central part of our lives, yet they are still far from accessible to the blind. This is especially unfortunate as such environments hold great potential for them for uses such as social interaction, online education and especially for use with familiarizing the visually impaired user with a real environment virtually from the comfort and safety of his own home before visiting it in the real world. We have implemented a simple algorithm to improve this situation using single-point depth information, enabling the blind to use a virtual cane, modeled on the “EyeCane” electronic travel aid, within any virtual environment with minimal pre-processing. Use of the Virtual-EyeCane, enables this experience to potentially be later used in real world environments with identical stimuli to those from the virtual environment. We show the fast-learned practical use of this algorithm for navigation in simple environments.

## Introduction

Imagine yourself immersed in a walk through Google-Earth learning your way to a place you have never been to, engaged in a first-person quest, or in the middle of a social event on Second-Life, when suddenly the lights go off. Deprived of visual information you can but stare at the screen in anguish, unable to participate in the virtual environment, which is based mainly on visual cues.

Virtual worlds and environments can be used for a wide range of useful and necessary purposes ranging from games through education and scientific research to simply general social interaction. Approximately ***onebillion*** people have experienced such virtual environments, and more than a ***quarterofabillion*** actively participate within one on a regular basis [1–3]. Since transfer of orientation and mobility knowledge for the blind between virtual and real environments is well established [4–6], these worlds hold even greater potential for the blind than for the general public. Familiarization with an environment in a virtual-environment, such as Google-Earth for example, is safer than learning to navigate it in the real world and risk getting hurt [7] or lost [8], and as this can be done alone at one’s own leisure spares the cost and availability problems of personal trainers.

Yet, virtual environments are currently barely accessible to the blind, as they are designed mainly around visual contact with the user, and standard tools such as screen-readers for making computerized information accessible to the blind are not designed for this purpose. This problem has birthed many attempts at making virtual worlds accessible to the blind, such as the HaptiGuide or BlindAid among many others [6,9–12], mainly attempting to simulate the use of the White-Cane virtually. Some environments were focused on an enjoyable or educational experience [6,13], while others on teaching the blind the outline of an environment and familiarizing them with it before a visit. However, none of these approaches has graduated past the research stage (see review in [14]).

These environments have mainly been limited by the extensive pre-processing required and were therefore not applicable to the leading online worlds, nor were they applicable on a large scale basis. Additionally, the input the user receives within them is very different from the one he will receive when navigating in the real world, as the stimuli within the simulation is very different from the tactile feeling of the white cane in use when detecting an obstacle or feeling the ground.

Furthermore, while even perfectly simulating the traditional white-cane in a virtual world enables studying an environment in a manner similar to real-world situations [5], it does not improve the way a blind person can learn or navigate an environment in the real or virtual world beyond the inherent current limited perception the white cane offers. The white cane is limited as it only enables perception of the environment at ground level and at a maximum of 1.2m distance. Obstacles at eyelevel, for example, cannot be detected and subsequently avoided with the use of the white cane alone, causing a great many injuries to the blind users in the real world [7].

In order to maximize the full potential of virtual environments for teaching and perceptual purposes we incorporated into the virtual world a more advanced mobility aid developed in our lab. We modeled this virtually simulated device upon the EyeCane virtual cane [15]. The EyeCane attempts to augment the classic white cane using sensors to detect obstacles from a greater distance (5m) and transforms this information into a simple auditory cue (the user hears a series of beeps, where the closer the object the user is pointing at, the higher the frequency of cues). The **Virtual-EyeCane**, which we describe here, gives the same sensory output as the real-world EyeCane. This feature has the potential of easing the transfer of learning from the virtual environment to the real one. Additionally, as this simulation relies only on the distance between the participant and the object he is pointing at, it can be easily calculated in any 3D mesh which is the foundation of every virtual environment.

In this research we investigate whether blind and blindfolded users can navigate down a virtual twisting corridor using only the Virtual-EyeCane’s single point distance parameter, after only a few minutes of training.

## Methods

### Ethics

**Figure 1:**
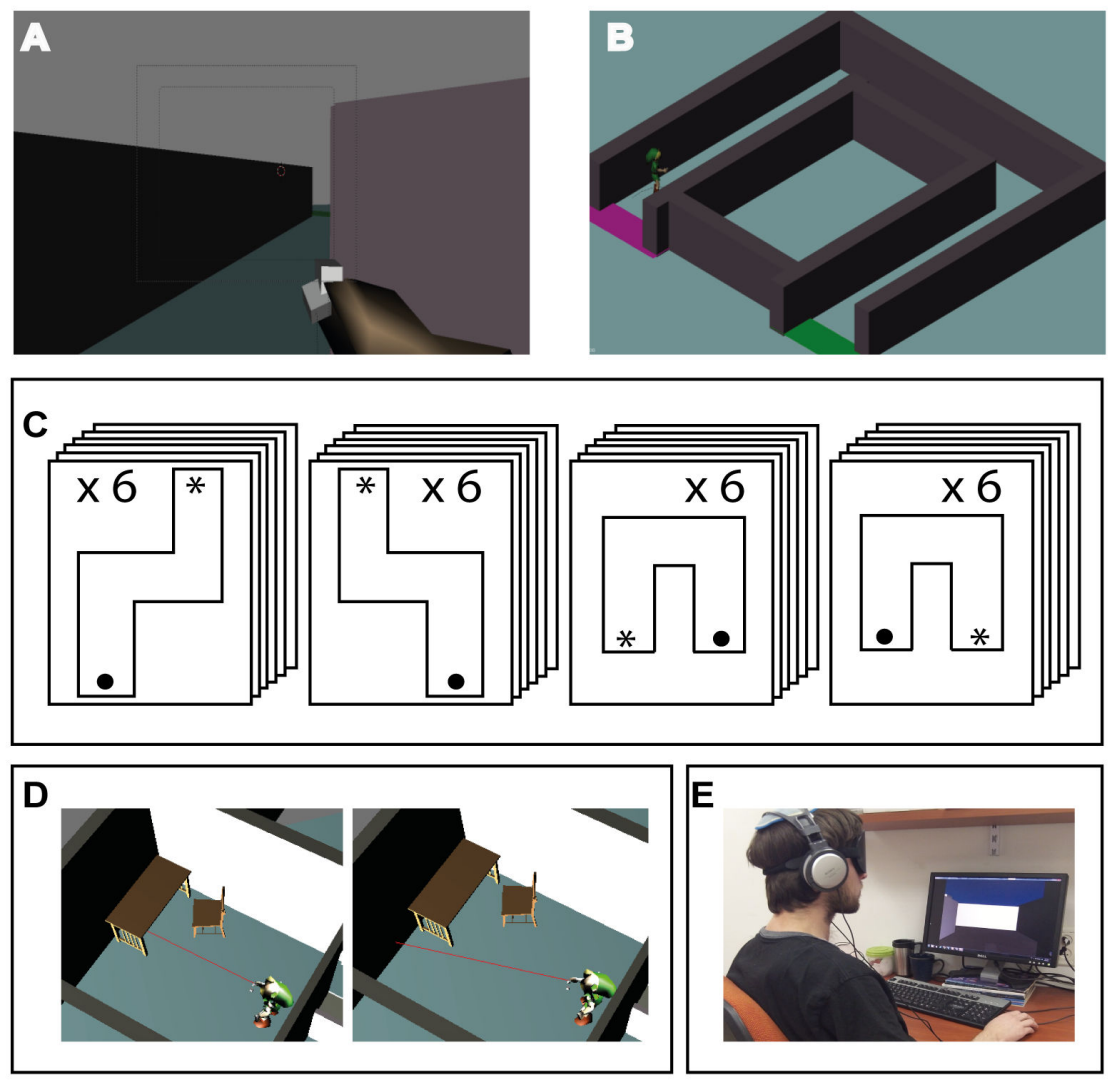
Experimental setup and paradigm. **A**. First person view of the device in a virtual environment **B**. Third person view of the device in a virtual environment **C. Experimental paradigm**: Diagrams of the 4 different levels, all equal in length and each including two 90^°^ turns. Each level was repeated consecutively 6 times, totaling 24 trials per participant. The internal-order between the four levels was determined pseudo-randomly. **D**. Illustration of the algorithm for the device: the user points the device at a location, the distance to that location is calculated and returned to the user, the user moves the device to a new location E. Picture of a blindfolded participant with the experimental setup.

This experiment was approved by the Hebrew university ethics committee in accordance with the 1964 Helsinki Declaration, and all participants signed informed consent forms. The participant in Figure 1E has given written informed consent, as outlined in the PLOS consent form, to publication of their photograph.

### The Virtual-EyeCane and virtual environment

The virtual environment in this experiment was created using Blender 2.49, and Blender-Python modules using python 2.6.2. The location and orientation of the user’s avatar and Virtual-EyeCane was tracked at all times at a rate of 60 Hz (identical to the rate of logic-ticks in the virtual environment, thus covering any possible in-game activity) to enable recreating and analyzing the participants’ route, collisions and time in each corridor. The environments have a graphical output to the screen, which was used by the experimenter to track the participants’ progress (as can be seen in Figure 1a & 1b. the participant always experiences the environments in 1^st^ person). Distances within the environment are set so that each “blender meter” correlates to a real world meter. Thus, the output from the Virtual-EyeCane at one “virtual meter” is the same as the output from the real-world EyeCane at a distance of one meter.

### The distance-to-sound transformation

In the real world, the EyeCane uses a set of IR sensors to get the distance to the object it is pointed at. This distance is then transformed to sound such that the shorter the distance the higher the frequency of sounds [15]. The Virtual EyeCane calculates the distance using Blender’s standard Ray-Casting algorithm (which calculates the distance to the object the virtual device is pointed at, much like the sensors of the EyeCane) and links it to a sound file recorded from the EyeCane’s auditory output (as we have done previously for 3D object recognition in [16]), which the user than uses to perceive the distance (see Figure 1d for illustration).

### Participants

23 participants (20 sighted & 3 congenitally blind, 9 Male, Aged 27.6±8.4). Sighted participants were blindfolded for the duration of the training and the test session.

### User interface

Participants were seated comfortably in front of a computer (see Figure 1e). Navigation was accomplished using the arrow keys on the keyboard. The participants were guided by the auditory feedback indicating their distance from the nearest object in front of them (in this case, the walls), a "success" cue (clapping hands) and a "collision" cue (recorded sound of a person colliding with a wall). The auditory cues were heard via standard headphones.

### Experimental procedure

Participants navigated in 24 trials, comprised of 4 different levels blocked in 6 consecutive repetitions of the same level (see Figure 1e). The order between the 4 level-blocks was selected pseudo-randomly and varied between participants. Participants were instructed to navigate through the routes to their end as quickly as possible, while avoiding collisions. No verbal feedback was given during the test phase, and participants were not told anything about them (especially the fact that corridors can, and do, repeat). After each level was completed participants were requested to draw the route they had taken. As in the "EyeCane" experiments [15] participants were instructed to focus on completing the corridors as swiftly as possible while minimizing the amount of collisions. Participants were given the option of "giving up" on a level and thus failing it and moving on to the next level.

### Training

Prior to the task participants travelled through 3 virtual training routes (a straight corridor, a left turn and a right turn) to familiarize them with the keyboard controls and environment. These routes were half the length of the regular routes, and participants were instructed and encouraged to experiment within them by walking back and forth in front of walls – so they become familiar with the auditory feedback associated with approaching a wall - and colliding with the walls while taking their time to explore the corridors. This training session lasted no more than 7 minutes and was accompanied by verbal feedback from the instructor.

### Statistical analysis

All p-values denote standard paired t-tests, besides the comparisons between the sighted and blind groups in which a non-paired t-test was used. SD is given together with mean results in the following format "Mean±SD". As the number of blind participants was small, statistical analysis was performed on the sighted only, and the blind results were compared to them.

## Results

### General results

All Sighted participants were able to complete all levels with an average time of 96±26.7 seconds, an average of 2.5±1.2 collisions, and an average distance of 14±1.7 virtual meters.

**Figure 2:**
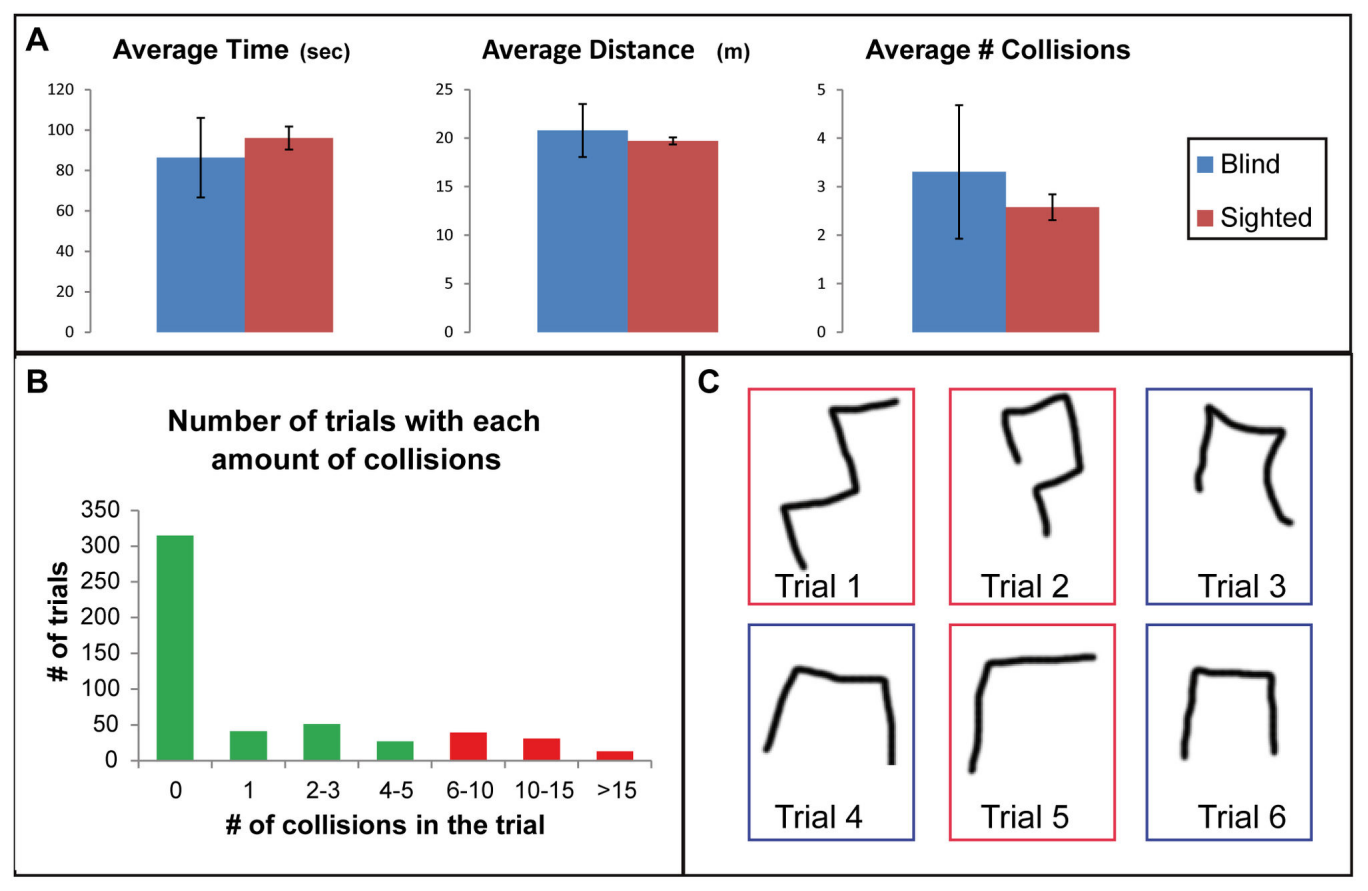
General results. **A**. Demonstrating the similarity of the results of the blindfolded-sighted and blind participants. **B**. The drawings of participant OL in 6 trials within the same level (the drawings of trials 3, 4, 6 are correct, and marked with blue frames). These drawing demonstrate that she did not perceive the repetition of the level. **C**. The number of trials with each number of collisions, demonstrating that the large majority of trials had 0 collisions.

All blind participants completed all levels. Their scores were similar to those of the sighted with an average time of 86.3±60.7 seconds (p=0.3 when compared to the sighted), average distance of 14.8.1±7.4 virtual meters (p=0.37) and average number of 3.3±5.3 collisions (p=0.22) as can be seen in Figure 2a.

Interestingly, participants did not report understanding that the trials repeated themselves. Their lack of knowledge of this fact is strengthened by their drawings of the routes they traveled through (See Figure 2b) which clearly show the different perception of different identical levels.

**Figure 3:**
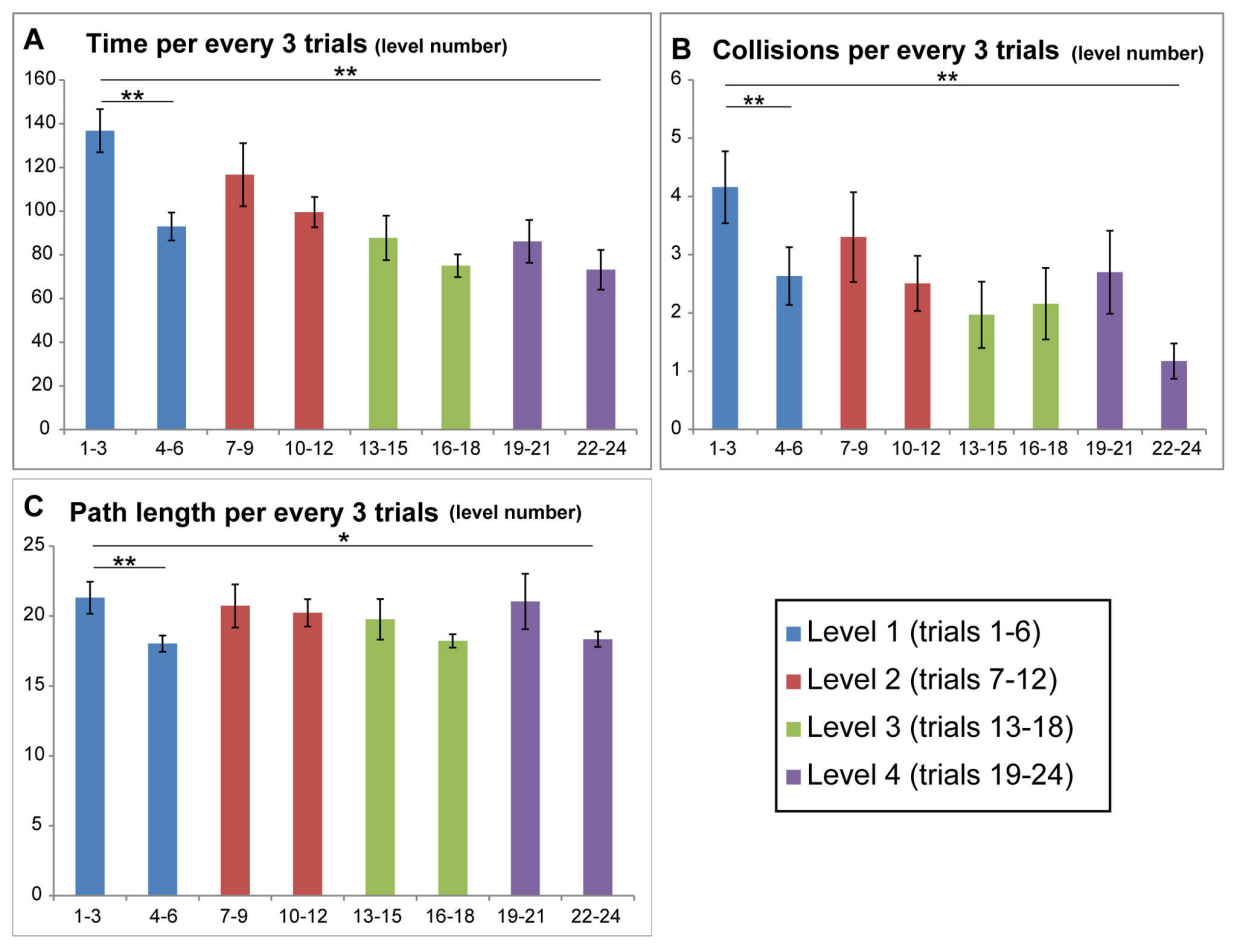
Average Time, Collisions & Path length. All graphs show the results with trials grouped in 3 such that the first 3 trials of each level and the last three trials are grouped, creating 2 columns per level. Each level has its own color. In all graphs both a significant general improvement trend and a non-significant within-level improvement trend can be see (**A**) Average time per every 3 trials (**B**) Average number of collisions per every 3 trials (**C**) Average length of path per every 3 trials.

For each parameter, two trends were evident: The first is a steady improvement throughout all levels. The second, which in many cases was observed as a trend but without statistical significance, is a worsening of performance in the first trials each time a new level was introduced, and an increasingly quick recovery time and improvement in the trials within each block. To demonstrate this, we will present the data such that each level is split into two halves, the first three identical trials of the level will be grouped as one and the second three will be grouped in the other, for each of the three performance parameters (Figure 3).

### Time

Participants improved significantly along trials from an average of 136.7±78.8 seconds to 73.1±72.8 seconds (p<7.9E-7 when comparing trials 1-3 and trials 21-24). The graph in Figure 3a clearly shows that whenever the participants encountered a new route their time worsened, though not significantly so.

### Collisions

Participants improved significantly along trials from an average of 4.1±4.9 collisions to 1.1±2.4 collisions (p<2.6E-5 when comparing the 1^st^ 3 trials to the final 3 trials). Note that as can be seen in Figure 2c there was large variance among participants and trials: 62.3% of the trials had 0 collisions, and an additional 8.1% had a single collision, while only 6% had more than 10 collisions. Thus, despite the average of 1.1±2.4 collisions for trial, most trials did not contain collisions.

The graph in Figure 3b shows that whenever the participants encountered a new route their number of collisions increased. However, these increases were not in a significant manner.

### Path length

As the required path length was set and equal for all levels, participants showed only a relatively small, though significant, improvement throughout the experiment (from 15.2±6.5 to 13.1±3.1, or 2.1m per trial, p<0.02 when comparing the 1^st^ 3 trials to the final 3 trials). While not as significant as the other parameters, the "jump" in participant’s route length when encountering novel routes is clear in Figure 3c as well.

## Discussion

### General

The results clearly show that all participants both sighted and blind, were able to navigate all routes successfully following minimal training. These results suggest that the approach of using virtual canes in virtual environments is both feasible and beneficial to potential users, and enables them to perform virtual tasks which are otherwise impossible for them.

Our results show that even using such a simple feedback parameter as distance to an object, and even using ***only*** it, can enable the blind to perform tasks otherwise impossible to them such as virtual navigation, improving the accessibility of online 3D environments and objects in a way that can easily be combined with other state-of-the-art tools for accessing other types of computerized information such as screen-readers.

A key point of our method is that it is easily usable without the need to computationally pre-process a specific scene, but rather relies on data that a customized plugin to almost any virtual environment will be able to supply. Thus, instead of having to create each scene with a virtual world, the user would only need to download a plugin for the specific environment (such as WoW, Google-Earth etc.).

Another factor enhancing our method’s accessibility is that it does not rely on any special equipment, but rather only on standard hardware – a sound output source (speaker/headphones) and input devices (mouse/keyboard).

### Comparison to other devices

Many other attempts to make virtual environments accessible to the blind have been made [9,11,14]. Most of these environments attempt to offer the blind representations of the real world for orientation and mobility practice before visiting novel sites. Our advantage over these tools is two-fold. First, our extendibility into existing virtual environments, and second, the Virtual-EyeCane enables the user to receive identical input in both the real and virtual environment, and thus creates a far more similar experience for the user.

At the same time, while some information is better than none, the information currently offered by the Virtual-EyeCane is still limited in scope, as a user of the Virtual-EyeCane receives no information about other visual parameters beyond distance (such as patterns or color). For example, while a Virtual-EyeCane user can easily locate an open door, a closed door will pose a difficult challenge which the device cannot aid in (it would sound like the continuation of the surrounding wall). This problem could be potentially solved by using the Virtual-EyeCane in combination with other sensory substitution devices (SSDs) such as the vOICe [17], TDU [18,19] or the EyeMusic [20,21] which would offer additional information to the blind user such as shape and color.

### Gamification and positive user experience

Participants treated the Virtual-EyeCane experiments as games, and often requested to continue playing with them after the experiment ended (***all*** participants volunteered to participate in future experiments). This echoes previous successful reactions to other online game-based tools for the blind such as audio-doom [22], and demonstrates the efficacy of the current popular trend of using gamification for general research and education in the general public.

### The concept of distance and environment perception

The Virtual-EyeCane and the EyeCane, offer an interesting view on absolute distance, and the way the user perceives his environment. But beyond offering a glimpse at how the user perceives the world around him, they also change this perception. This is especially true for blind participants, whose perception of distance and space beyond their peri-personal space is limited. As one of our blind participants described the use of the "EyeCane": "I could feel the world stretching out before me. It was as if my hand could reach much further. As if the silent objects at the other side of the room were suddenly there" (Participant B2 [15]). This change in perspective may have an influence on the basic space-orientation networks in the brain, and it is intriguing to explore both if the blind represent space as the sighted do, and if not how learning to use these devices influences the neural basis of their perception.

Another interesting aspect of the user’s environment perception is the perspective offered to them – while most of the previous tools present 3rd person map type environments, offering an allocentric point of reference, using the Virtual-EyeCane leads to an egocentric mapping of the environment. This is significant as it has been shown repeatedly that the congenitally blind rely far more egocentric mapping methods than allocentric ones [23,24]. It would thus be interesting to explore whether offering this egocentric perspective improves their ability to learn new environments.

## Conclusions

We have shown here that using our method it is both feasible and simple to navigate down a simple twisting virtual corridor, for both blind and blindfolded-sighted individuals, a task which is not possible for them without such a method.

This method has several advantages such as simplicity and extendibility to existing virtual environments, thus increasing their accessibility to the blind. The use of identical stimuli in the virtual world from the Virtual-EyeCane to that given from the EyeCane in the real world may potentially increase the efficiency of navigation through a novel real-world environment learned virtually.

We believe that this method can be used in the future for enabling the visually impaired to accomplish further, more complex, tasks in virtual environments, and for making these environments more accessible in general. We believe this ability will be extended significantly when used together with other devices such as a virtual version of the EyeMusic.
